# Expression of a Leukocyte-Specific Antigen During Ontogeny in *Xenopus Laevis*

**DOI:** 10.1155/1991/60923

**Published:** 1991

**Authors:** Paula B. Smith, James B. Turpen

**Affiliations:** Laboratory of Developmental Immunology Department of Anatomy University of Nebraska Medical Center Omaha NE 68198-6395 USA

## Abstract

The monoclonal antibody CL21 recognizes a determinant present on the surface of leukocytes, but not on erythrocytes or nonhemopoietic tissue. The CL21 antigen was first expressed at 48 hr of development at 20°C (stage 28) on embryonic cells cultured from lateral plate mesoderm. Based on immunofluorescence staining and flow cytometric analysis, the distribution of fluorescence intensity of larval thymocytes and splenocytes was unimodal. Distributions of dull and bright cells were detected in both adult thymocytes and splenocytes. These different subpopulations appeared during the late perimetamorphic period. Adult splenocytes were metabolically activated when cultured in the presence of mAb CL21 bound to a substrate but not in the presence of mAb CL21 in suspension. Immunoprecipitation and SDS-PAGE under nonreducing conditions revealed that a single 180-kD molecule was expressed on thymocytes. Analysis of splenocytes demonstrated the presence of two molecules having similar molecular mass that resolved to a single band under reducing conditions.


*Developmental Immunology*, 1991, Vol. 1, pp. 295–307
Reprints available directly from the publisher
Photocopying permitted by license only

© 1991 Harwood Academic Publishers GmbH
Printed in the United Kingdom

### Expression of a Leukocyte-Specific Antigen During Ontogeny in *Xenopus Laevis*

PAULA B. SMITH and JAMES B. TURPEN*

*Laboratory of Developmental Immunology, Department of Anatomy, University of Nebraska Medical Center, Omaha, NE 68198-6395*

The monoclonal antibody CL21 recognizes a determinant present on the surface of leukocytes, but not on erythrocytes or nonhemopoietic tissue. The CL21 antigen was first expressed at 48 hr of development at 20°C (stage 28) on embryonic cells cultured from lateral plate mesoderm. Based on immunofluorescence staining and flow cytometric analysis, the distribution of fluorescence intensity of larval thymocytes and splenocytes was unimodal. Distributions of dull and bright cells were detected in both adult thymocytes and splenocytes. These different subpopulations appeared during the late perimetamorphic period. Adult splenocytes were metabolically activated when cultured in the presence of mAb CL21 bound to a substrate but not in the presence of mAb CL21 in suspension. Immunoprecipitation and SDS-PAGE under nonreducing conditions revealed that a single 180-kD molecule was expressed on thymocytes. Analysis of splenocytes demonstrated the presence of two molecules having similar molecular mass that resolved to a single band under reducing conditions.

KEYWORDS: Leukocyte common antigen, monoclonal antibodies, leukocyte development, *Xenopus*.

### INTRODUCTION

The *Xenopus* embryo is a unique model for studying early development of the hematopoietic and immune systems. Extensive study has shown that many aspects of the *Xenopus* hematopoietic and immune systems are similar to those of more recent vertebrates (Du Pasquier et al., 1989). Additionally, *Xenopus* embryos are accessible to experimental intervention at all developmental stages, making in vivo, physiological studies of the earliest events in hematopoietic differentiation possible.

In order to examine the parameters involved in stem-cell development in more detail, we have removed lateral plate mesoderm from stage 20 *Xenopus* embryos and placed it in culture (Turpen and Smith, 1985). Under these conditions, morphologically recognizable hematopoietic cells, primarily erythrocytes, monocytes/macrophages, and neutrophils differentiate after 5 days. However, a major limitation of these studies has been the use of morphological differentiation as an end point of the assay. Thus, we have been unable to examine the earliest events of hematopoiesis and have been unable to determine the time in development when lateral plate mesoderm begins to express the hematopoietic phenotype.

In this paper, we report on the development of a monoclonal antibody, CL21, which detects a 180-kD antigen expressed on the cell surface of leukocytes. The CL21 antigen is initially detected early in development, at stage 28 (approximately 48 hr postfertilization), and is stably expressed throughout the larval period. Experiments carried out using adult splenocytes show that binding of the CL21 determinant increases metabolic activity and suggests that CL21 may participate in either cell–cell or cell–extracellular matrix interactions.

### RESULTS

### Cell and Tissue Distribution

Flow cytometric analyses of cell suspensions from larval, perimetamorphic, and adult thymus, spleen and peripheral blood, stained with either

*Corresponding author.

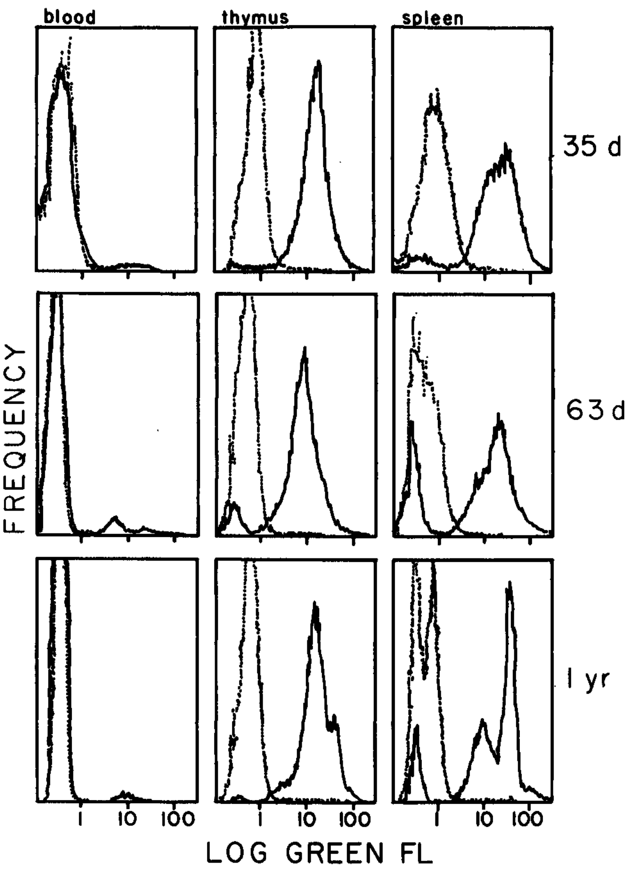

FIGURE 1 Flow cytometric analysis of CL21 expression. Thymus, spleen, and peripheral blood populations were analyzed for CL21 expression at 35 days (stage 55), 63 days (stages 65–66), and 1 year postfertilization. Dotted lines represent fluorescence intensity after staining with an isotype control antibody and solid lines represent fluorescence intensity after staining with mAb CL21. $3\times10^4$ cells were analyzed from each population.

mAb CL21 or isotype control mAb 76-2-11 ($IgG_{2ak}$) are shown in Fig. 1. All hematopoietic cells in the thymus stained with mAb CL21. Larval thymocytes from 20-, 35- and 42-day-old animals showed a unimodal distribution of fluorescence intensity, but adult thymocytes exhibited a tripartite distribution. The majority (70%) of thymocytes displayed an intermediate fluorescence intensity, while 20% were more intense and 10% were dull. Only the intermediate subpopulation was detectable prior to the completion of metamorphosis. Larval splenocytes exhibited a unimodal distribution in fluorescene intensity. A bimodal distribution was observed in the adult; 65% of positive splenocytes were stained brightly. These subpopulations became detectable near the completion of metamorphosis. Although we were able to discriminate two splenocyte and three thymocyte subpopulations in all frogs examined ($n$=5), the proportion of dull and bright cells varied with each individual. Five percent of cells in larval peripheral blood stained with the antibody. The percent of positive cells in adult peripheral blood decreased to 2%. The proportion of peripheral blood cells positive for mAb CL21 corresponded to WBC counts in peripheral blood of larvae and adults. There was no staining of either larval or adult preparations with an isotype control antibody specific for swine cytotoxic T-cells (mAb 76-2-11).

In order to determine the identity of the cells negative for mAb CL21, adult splenocytes were stained and sorted into CL21 positive and CL21 negative populations. Resulting suspensions were cytospun and examined for morphology (Fig. 2). Thrombocytes were identified using the periodic acid-schiff reaction [PAS] (Hadji-Azimi et al., 1987; Hayhoe and Quagline, 1988). Panel 2A shows the fluorescence profile of unsorted splencocytes. Erythrocytes, leukocytes (2D), and PAS positive thrombocytes (2G and H) were identified in this population. CL21 positive cells (2B) were identified morphologically as leukocytes and did not contain PAS positive cells (2E). On the other hand, CL21 negative splenocytes (2C) were identified as erythrocytes (2F) and PAS positive thrombocytes (panels 21 and 2J). Thus, the CL21 negative cells seen in the spleen and peripheral blood (Fig. 1) include mature erythrocytes and thrombocytes. The remaining leukocytes are CL21 positive.

Tissue distribution of CL21 in adult frogs was determined by staining frozen sections with

FIGURE 2 Erythrocytes and thrombocytes are CL21 negative. Adult splenocytes were sorted into CL21 positive and CL21 negative populations on the basis of fluorescence intensity. Each cell population was characterized by morphology and cytochemistry (PAS reaction). Panels A, B, and C represent fluorescence profiles of unsorted, $CL21^+$, and $CL21^-$ splenocytes, respectively. Panels D, E, and F represent Giemsa stained cytospins from unsorted, $CL21^+$, and $CL21^-$ splenocytes, respectively. Panels G and I are phase-contrast photomicrographs, and panels H and J are bright field photomicrographs of cytospins after the PAS reaction. Matching arrows identify $PAS^+$ thrombocytes in identical fields from unsorted (G, H) and $CL21^-$ (I, J) preparations. Bar=10 microns.

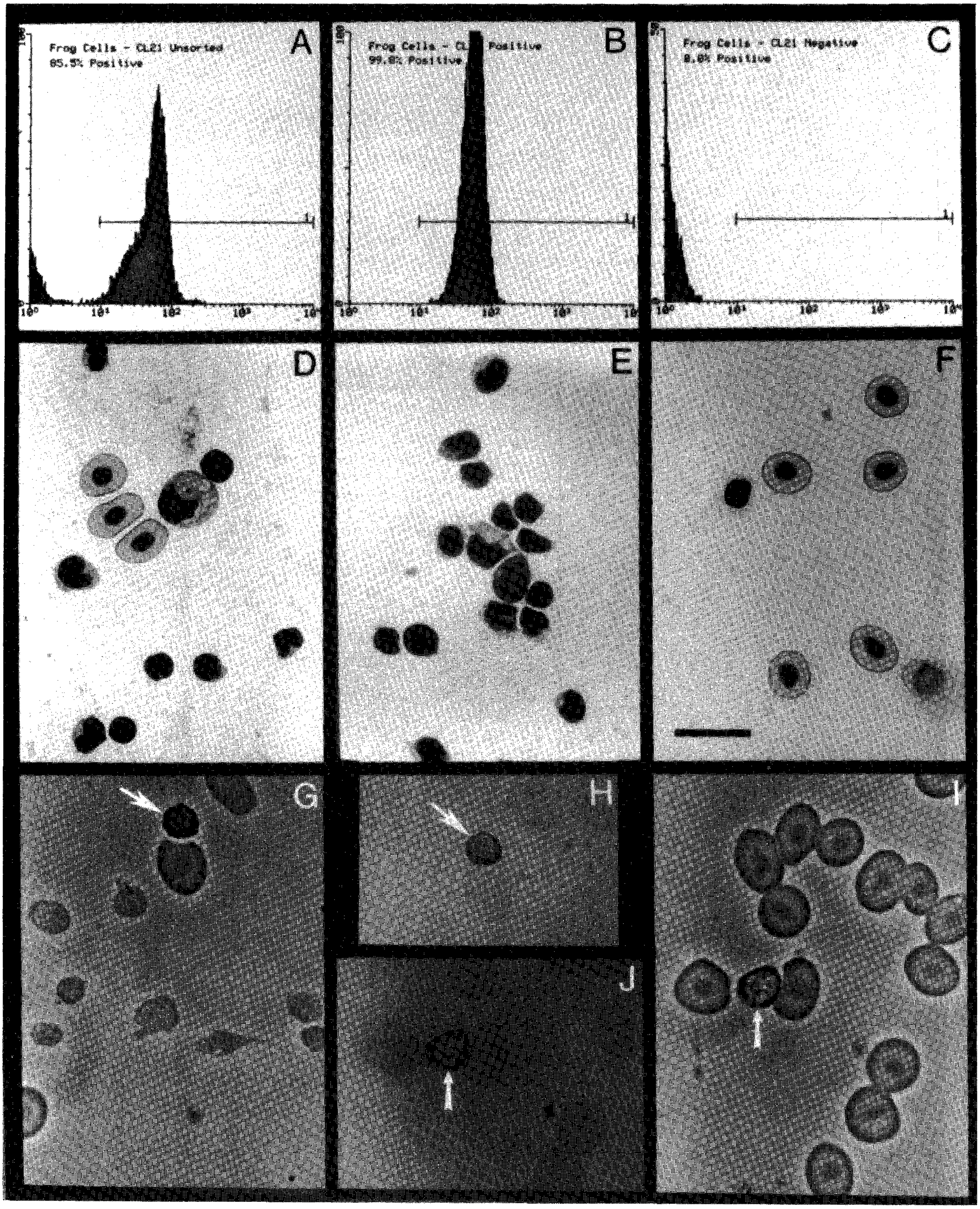

A

Frog Cells - CL21 Unsorted

85.5% Positive

B

Frog Cells - CL

Positive

99.8% Positive

C

Frog Cells - CL21 Negative

Positive

D

E

F

G

H

I

J

FIGURE 2

mAbs CL21 and AM20 (data not shown). The thymic cortex stained brighter than the medulla. There were focal accumulations of positive cells in the perihepatic layer of the liver. Additionally, mAb CL21 stained isolated cells within the dermal layer of the skin. The CL21 antigen was not expressed in heart muscle, gut epithelium, or kidney parenchyma. This distribution of the CL21 antigen coincided with the distribution of MHC II determinants recognized by mAb AM20, except that AM20 also stained isolated cells in the kidney parenchyma and gut epithelium.

### Molecular Weight Characterization

Surface proteins were radiolabeled with $^{125}I$ and the antigen recognized by mAb CL21 was immunoprecipitated from lysates of adult thymus and spleen. Figure 3 shows the results after separation on polyacrylamide gels under reducing and nonreducing conditions. Under nonreducing conditions, the CL21 antigen from the thymus (lane a) migrated as a single band with a relative Mr between 160 and 180 kD. The CL21 antigen immunoprecipitated from splenic lysates (lane b) was represented by two distinct bands within the same molecular-weight range. (However, the molecular weight of the upper band may not be accurate because the radioactive band appears to be distorted by the unlabeled IgG.) Under reducing conditions, a major and a diffuse band were evident in precipitates from the thymus (lane c), but splenic (lane d) antigens migrated as a single band. When the CL21 determinant was immunoprecipitated from 20-, 35-, and 42-day-old larval thymuses and spleens, no age-related differences in electrophoretic mobility were observed (data not shown).

Figure 4 shows the results of an experiment to confirm that the antigen recognized by mAb CL21 was absent from erythrocytes. Adult peripheral blood was fractionated over a ficoll gradient to remove the majority of leukocytes. Cell suspensions of thymus, spleen, and purified erythrocytes were radiolabeled with $^{125}I$, immunoprecipitated using mAb CL21, and analyzed under reducing conditions. The presence of the 160–180-kD doublet was confirmed in the thymus (lane a) and as well the single 180-kD band in the spleen (lane b). No evidence of a surface molecule recognized by mAb CL21 was detected in erythrocyte lysates (lane c).

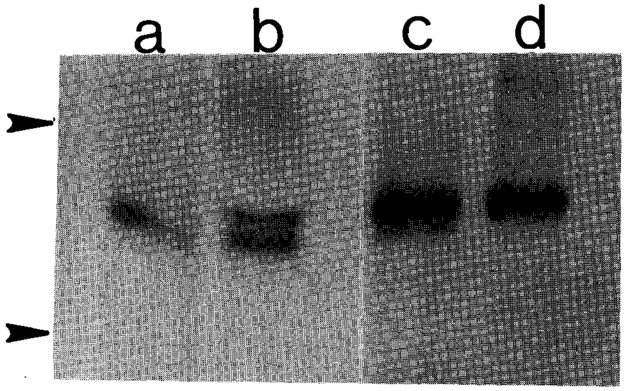

FIGURE 3 Molecular-weight characterization of CL21 determinant. Immunoprecipitates from radiolabeled adult thymus (lanes a and c) and spleen (lanes b and d) were analyzed by SDS-PAGE under nonreducing (lanes a and b) and reducing (lanes c and d) conditions. Positions of 200 and 100-kD molecular-weight markers are indicated by arrows.

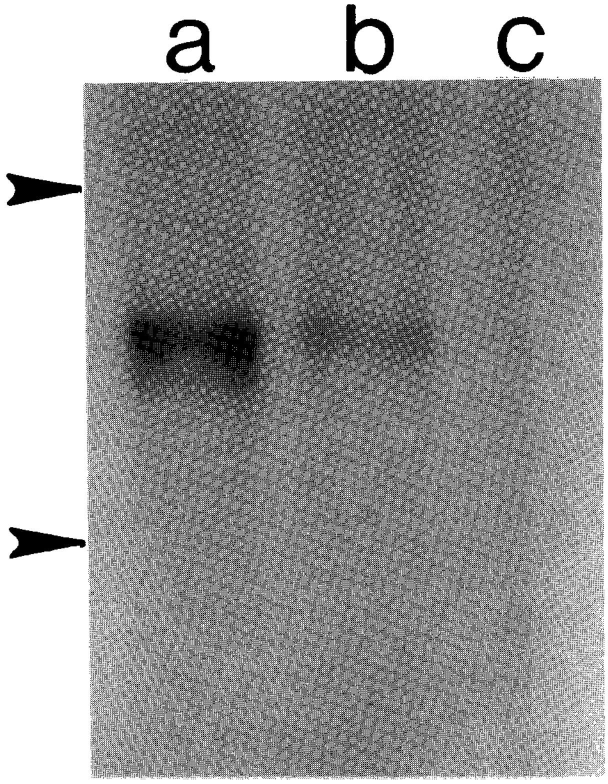

FIGURE 4 CL21 is not expressed on erythrocytes. Immunoprecipitates from radiolabeled thymus (lane a), spleen (lane b), and ficoll purified peripheral blood erythrocytes (lane c) were analyzed by SDS-PAGE under reducing conditions. Arrows indicate position of 200 and 100-kD molecular-weight markers.

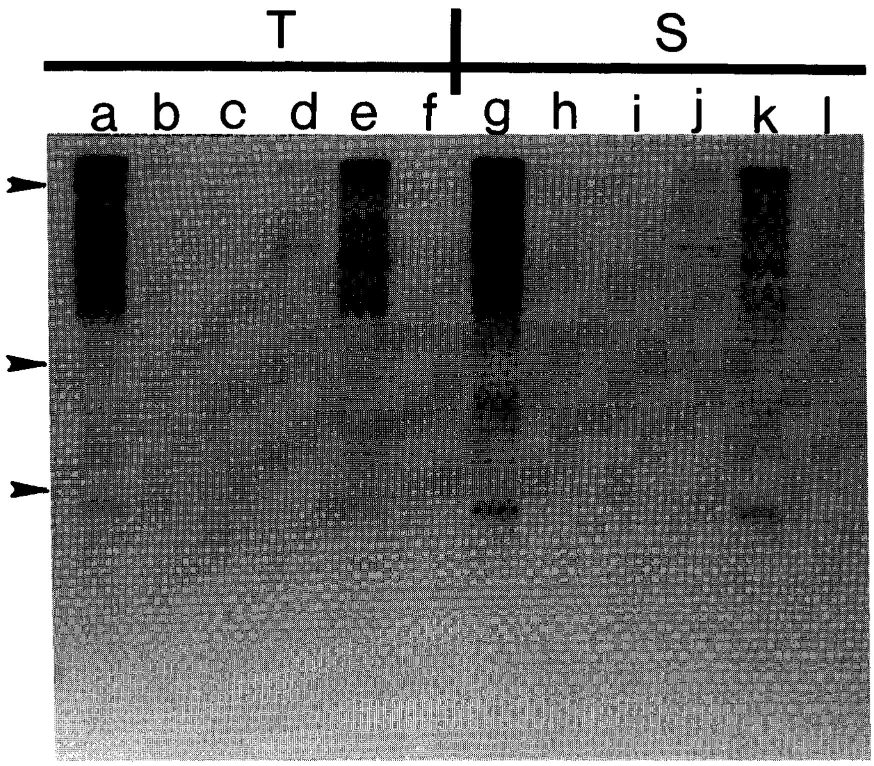

FIGURE 5 The determinant recognized by mAb CL21 is a subset of the determinants recognized by mAb XL-1. Radiolabeled adult thymocytes (T) and splenocytes (S) were precleared with either XL-1 (lanes b, c, h, and i) or CL21 (lanes e, f, k, and l). Lanes a, b, e, g, h, and k were immunoprecipitated with mAb XL-1. Lanes c, d, f, i, j, and l were immunoprecipitated with CL21. Arrows indicate position of 200, 100, and 69-kD molecular-weight markers analyzed under reducing conditions.

Recently, Ohinata et al. (1989) have reported the development of mAb XL-1, which recognized determinants present on *Xenopus* leukocytes but not erythrocytes. Western blot analysis of the XL-1 determinants revealed several molecules ranging from 180–250 kD in thymocytes and splenocytes, but none in erythrocytes. In contrast to CL21, XL-1 reacted with thrombocytes as well as leukocytes. In addition, mAb XL-1 reacted with serum components (35–200 kD). Therefore, it was important to establish whether mAbs CL21 and XL-1 recognize the same determinant. Figure 5 shows the results of an experiment in which thymocytes and splenocytes were radiolabeled with $^{125}I$ and surface molecules were analyzed using these two antibodies. Immunoprecipitations from thymus and spleen using mAb XL-1 are shown in lanes a and g. Under these conditions, this antibody recognized a family of cell-surface determinants ranging from 200 to 65 kD. Lanes b and h show the results when thymic and splenic lysates were first precleared with mAb XL-1 followed by immunoprecipitation with mAb XL-1. These data demonstrate that all molecules recognized by the mAb XL-1 were removed by preclearing. Lanes c and i show the results when lysates were precleared with mAb XL-1 and immunoprecipitated with mAb CL21. Note that preclearing with mAb XL-1 removed all activity precipitable with mAb CL21. The complementary experiment is shown in lanes d, e, f for thymus and j, k, l for spleens, respectively. Lane d is an immunoprecipitation from the thymus using mAb CL21. Lane e represents thymic lysate that was precleared with mAb CL21 and immunoprecipitated using mAb XL-1. Note that the 180-kD band present in lane a is absent from lane e. Lane f represents both preclearing and immunoprecipitation with mAb CL21. Similar results were seen with the spleen. Therefore, the molecule(s) recognized by mAb CL21 belongs to a family of molecules recognized by mAb XL-1.

### Splenocyte Activation in the Presence of mAb CL21

We wished to determine what effect, if any, mAb CL21 had on splenocyte activation as colormetrically detected using reduction of the tetrazolium salt MTT as an indicator. The MTT assay, which

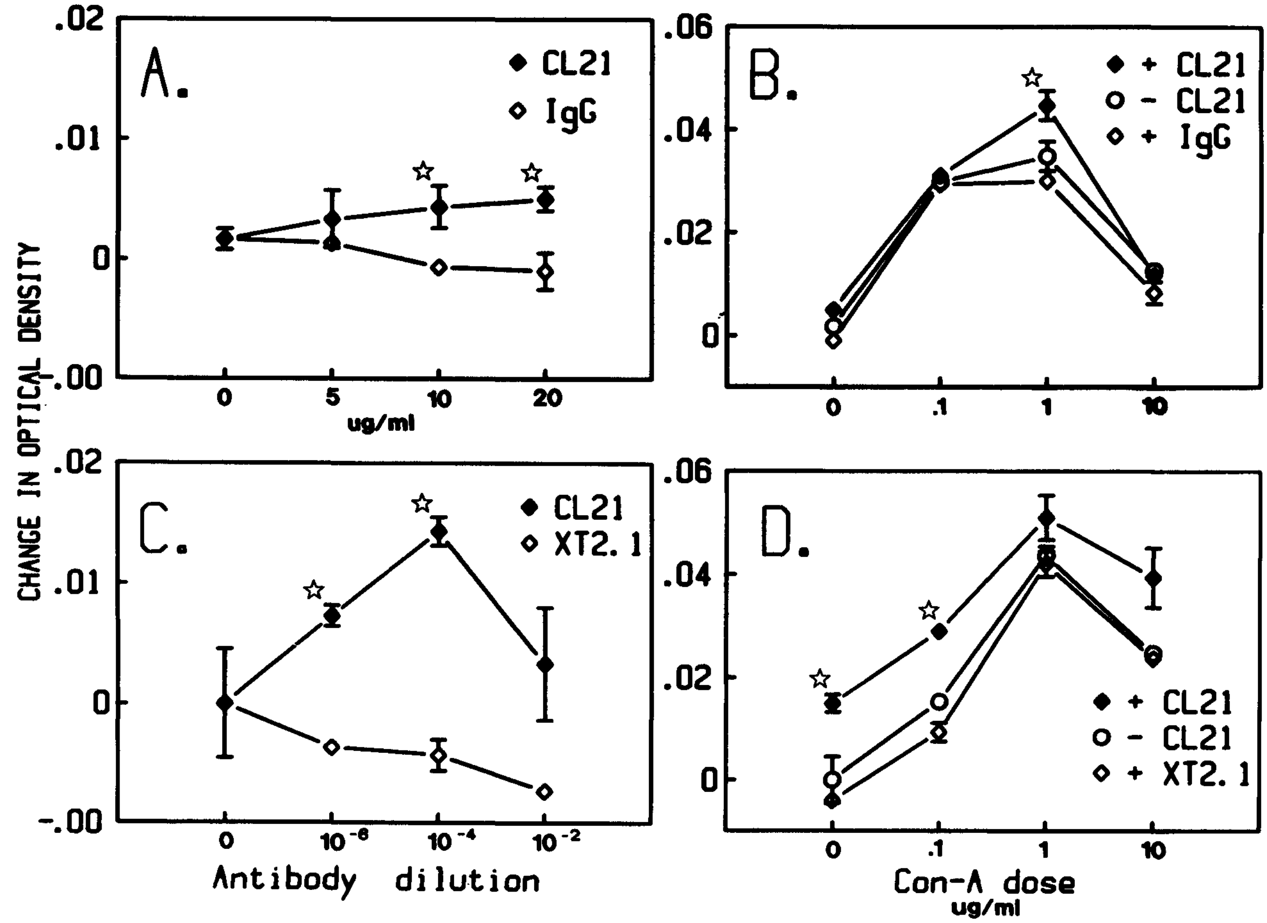

FIGURE 6 Metabolic activation of adult splenocytes by mAb CL21 bound to a substrate. (A) Dose response to affinity-purified CL21 or isotype control (IgG). (C) Dose respose to CL21 or XT-2.1 ascites. (B and D) Augmentation of Con-A response (0, 0.1, 1, and 10 $\mu$g/ml) in the presence of CL21, either affinity-purified (20 $\mu$g/ml) or ascites ($10^{-4}$ dilution). Each point represents mean± s.e. of three replicates. ☆ Indicates points of statistical difference ($p<0.05$) using Student's $t$ test for small samples. Graphs are representative of five separate experiments.

detects live, metabolically active cells, is useful for measuring cytoxicity, proliferation, or activation since the degree of signal generated depends on the degree of cellular metabolic activity (Mossman, 1983). Adult splenocytes were cultured in the presence of different concentrations of mAb CL21 attached to 96 well flat bottom plates via goat antimouse IgG. As can be seen in Fig. 6A and 6C, a dose-dependent metabolic activation was observed in the presence of either affinity-purified mAb CL21 or mAb CL21 ascites, but not in the presence of affinity-purified mAb 76-2-11 or mAb XT2.1 ascites. It should be noted that metabolic activation by mAb CL21 did not lead to a detectable increase in DNA synthesis as measured by $^3$H-thymidine incorporation (data not shown). Figures 6B and 6D show the effect of immobilized mAb CL21 on the splenocyte response to the T-cell mitogen Con-A. An enhancement of the Con-A response was seen in the presence of mAb CL21, but not in the presence of mAbs 76-2-11 or XT2.1. When these antibodies (CL21, XT2.1, 76-2-11 or goat antimouse IgG) were used in suspension, these specific

FIGURE 7 Developmental expression of CL21 determinant on hematopoietic precursors. Phase contrast (A, C, E) and fluorescence (B, D, F) photomicrographs of cells from cultures of lateral plate mesoderm. A–D were from 1.5-day-old cultures (control embryos at stage 27/28) and E and F were from 2-day-old cultures (control embryos at stage 35). B was stained with isotype control antibody and D and F were stained with mAb CL21. Bar=10 microns; arrows show same cell in each field.

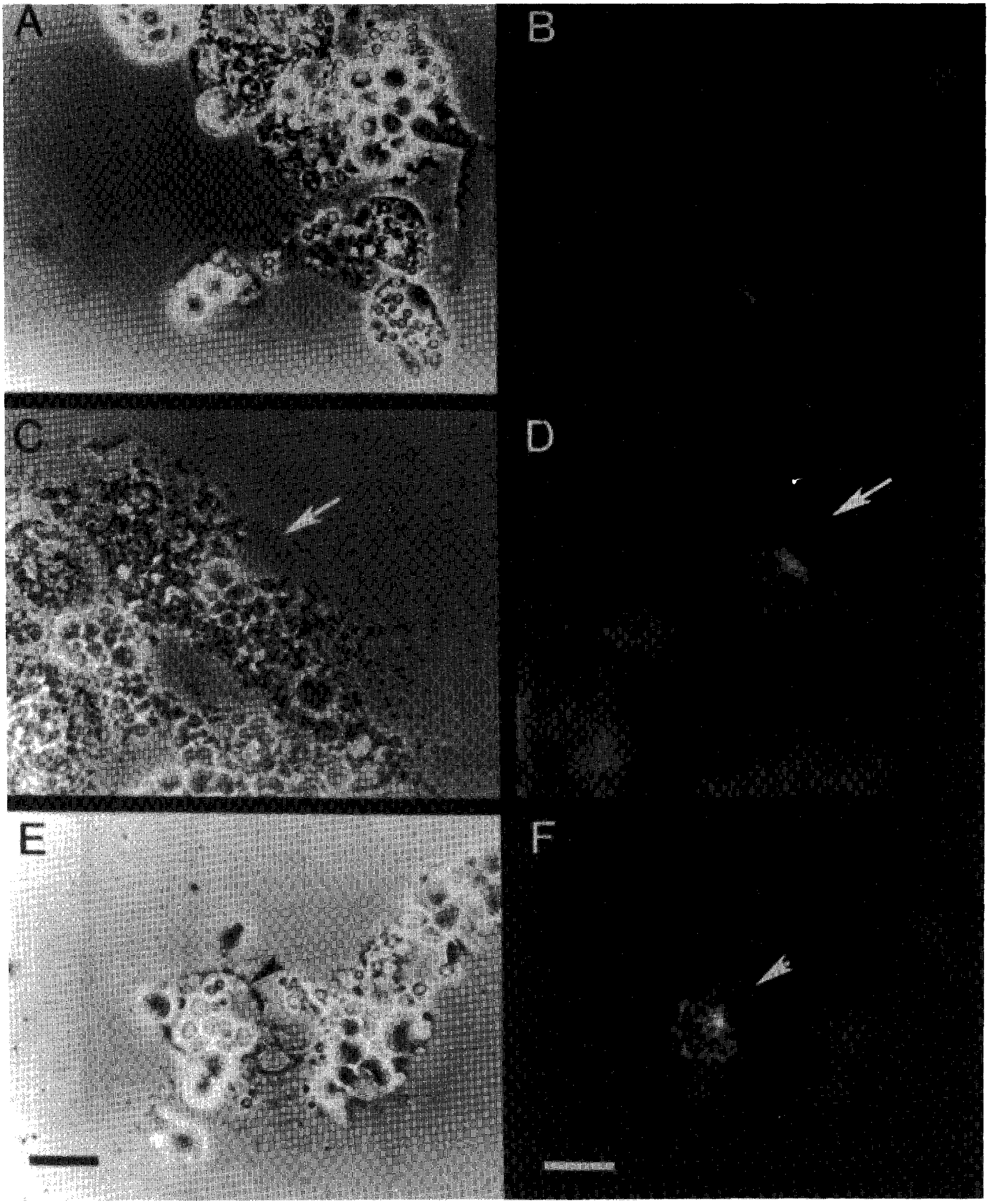

FIGURE 7

dose-dependent responses were not observed (data not shown).

### Developmental Expression of the Antigen Recognized by mAb CL21

Finally, we wanted to determine the time during ontogeny when CL21 antigen expression begins. Representative blastulae, gastrulae, neurlae, and tailbud embryos (stages 8, 11, 14, 19, and 23; Nieuwkoop and Faber, 1967) were dissociated in $Ca^{++}$- and $Mg^{++}$-free Steinberg's solution and stained with either mAb CL21, AM20, 6.16, or XT2.1. We did not detect expression of CL21 nor any other determinant at these early stages (data not shown).

At stage 19 (late neurulae), lateral plate mesoderm was removed from embryos and placed in culture for periods ranging from 1 to 10 days. Stage-matched control embryos were reared under the same time and temperature conditions as the cultures. At selected intervals, cultures were harvested and stained with either mAb CL21, XT2.1, 6.16, or AM20. Since mAb AM20 is the same isotype as CL21, this reagent also served as an isotype control. At no time during this experiment was staining with mAb XT2.1, 6.16, or AM20 detectable (Fig. 7A and 7B). CL21 expression was first detected in the perinuclear region of an isolated cell after 1.5 days in culture. Control embryos had reached stage 27/28 (3 hr after the beginning of muscular response) (Fig. 7C and D). At this stage, morphologically recognizable hematopoietic cells have not differentiated in culture and all cells are characterized by prominant yolk granules. After 2 days in culture, control embryos had reached stage 35 (hatching) and the number of CL21-expressing cells increased in both frequency and intensity (Figs. 7E and F). At this stage, morphological differentiation was still absent. Beginning at 2.5 days of culture, stage 37 for control embryos, cells expressing the CL21 antigen were widespread (Figs. 8A and B). During the following 4 days, control embryos reached stage 46 (feeding) and CL21-expressing leukocytes could be readily distinguished from non-CL21-expressing erythrocytes (arrows, Figs. 8C and D). Previous studies correlated benzidine positive erythroid cells with heterochromatic nuclear morphology and central nuclear location (Turpen and Smith, 1985). Thus, the antigen recognized by mAb CL21 is expressed by differentiating hematopoietic cells early in embryonic development prior to overt, morphological differentiation.

### DISCUSSION

The mAb CL21 recognizes a 160–180-kD molecule expressed on the surface of *Xenopus* leukocytes, but not on erythrocytes or somatic tissue. The antigen was not detected in embryonic tissues prior to 48 hr of development. Beginning at 48 hr, the antigen was expressed on differentiating lateral plate mesoderm, the embryonic tissue that gives rise to hematopoietic precursors. Functional studies have shown that binding of the antigen recognized by mAb CL21 increased metabolic activity of adult splenocytes and enhanced the splenocyte response to Con-A. Based on molecular weight and tissue distribution, we hypothesize that the CL21 determinant may be similar to members of the leukocyte common antigen family (CD45) described in mice and humans (Thomas, 1989).

Members of the CD45 family make up approximately 10% of the molecules on the lymphocyte surface and contain much of the carbohydrate moieties expressed by lymphoid cells (Tonks et al., 1989). Lymphocyte subpopulations can be delineated on the basis of distinct CD45 isoforms recognized by different monoclonal antibodies (Smith et al., 1986). Moreover, during maturation of prothymocytes to mature T cells, there is developmentally regulated expression of CD45 family members (Lefrancois and Goodman, 1987). For these reasons, we wanted to compare the antigens recognized by mAb CL21 with the putative leukocyte common antigen recognized by mAb XL-1 (Ohinata et al., 1989). It was thought that the antibodies might detect different isoforms within a CD45-like family and could therefore be useful for defining subsets of hematopoietic cells. However, our experiments suggest that the XL-1 antibody indiscriminately recognized determinants present on several cell-surface molecules. This pattern of reactivity suggests that mAb XL-1 may be directed against common carbohydrate epitopes. On the other hand, mAb CL21 recognized a unique subset of the antigens detected by mAb XL-1.

Under nonreducing conditions, mAb CL21 precipitated two bands from splenocytes and one

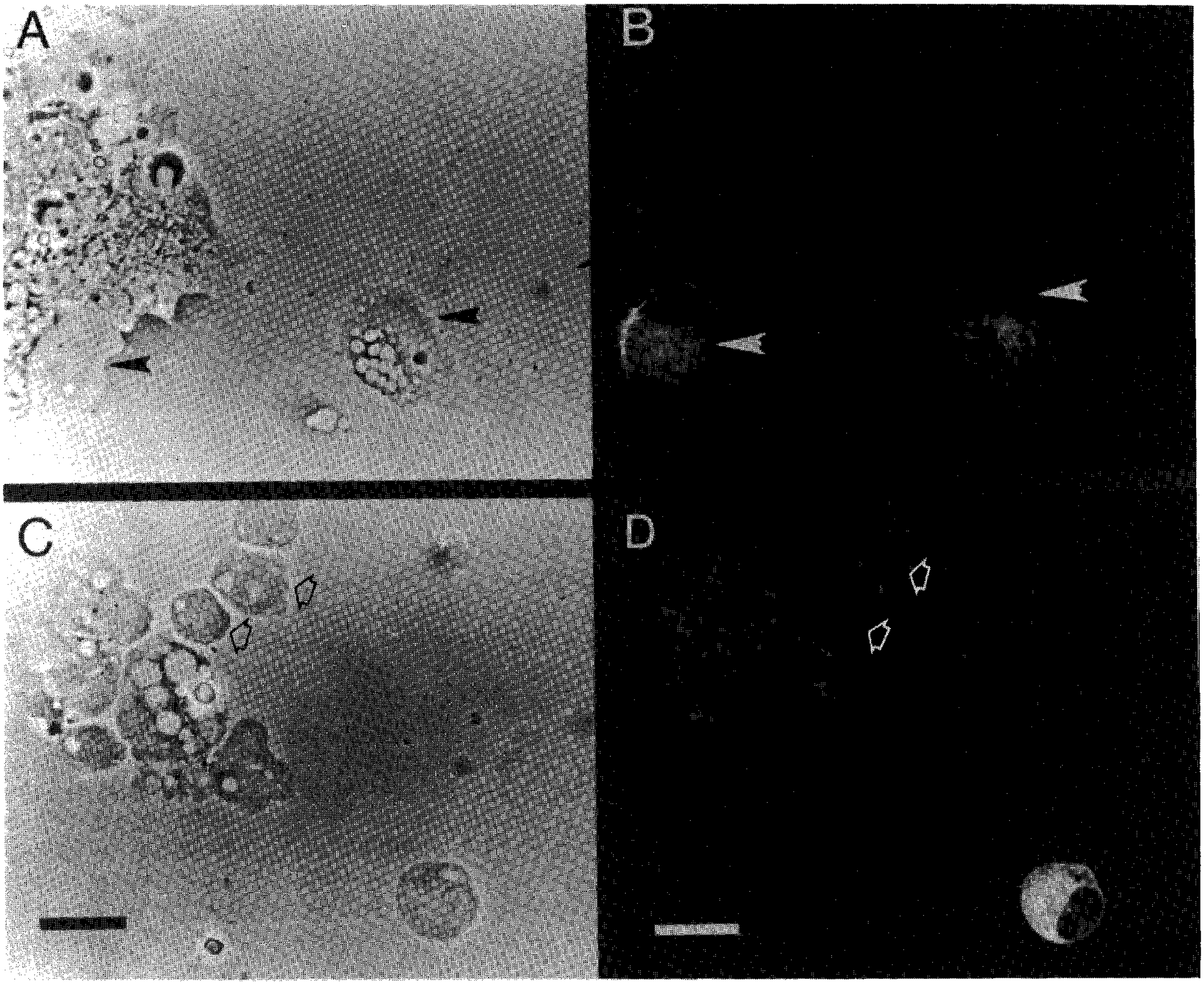

FIGURE 8 Developmental expression of CL21 (continued). Phase contrast (A and C) and mAb CL21 fluorescence staining (B and D) of cells from cultures of lateral plate mesoderm established for 2.5 days (A and B) and 7 days (C and D). Control embryos were at stages 37 and 46, respectively. Arrows in C and D indicate erythrocytes. Bar=10 microns.

major band from adult thymocytes. Under reducing conditions, the multiple splenocyte bands resolved into a single band, and an additional diffuse band appeared in thymocytes. These data suggest that *Xenopus* leukocyte subpopulations express similar high-molecular-weight molecules that may exhibit differences in tertiary structure when in their native, nonreduced configuration.

By using flow cytometric analysis, three subpopulations in the adult thymus could be distinguished on the basis of fluorescence intensity following staining with mAb CL21. In sections of the adult thymus, the cortex stained brighter than the medulla. However, during larval development, there appeared to be a single population of brightly staining cells. Whether fluorescence intensity will be useful for discriminating maturation sequences within *Xenopus* thymocyte subpopulations remains to be determined.

Depending on conditions, antibodies to CD45 have been shown to either augment or inhibit T-cell proliferation (Pingel and Thomas, 1989; Thomas, 1989; Samelson et al., 1990). Ledbetter et al. (1988) reported that CD45 regulates both signal transduction by lymphocyte-receptor molecules as well as T- and B-cell proliferation. Lymphocyte activation, defined as an increase in cytoplasmic-free calcium, occurs when either

CD2 or CD3 are cross-linked on the cell surface. This activation was inhibited when antibodies to CD45 were cross-linked with antibodies to CD3. On the other hand, lymphocyte activation induced by cross-linking CD4 was amplified when CD4 was cross-linked to CD45. Thus, CD45 can either up regulate or down regulate signal transduction, depending upon the cell-surface receptor with which it becomes associated. Since CD45 has been shown to be a receptor protein tyrosine phosphotase (PTPase) (Tonks, et al., 1988), these different metabolic affects may reflect different intracellular substrates for PTPase associated with CD2, CD3, or CD4. In the present experiments, we examined the effect of mAb CL21 binding on splenocyte activation. When mAb CL21 was immobilized on a substrate, a dose-dependent splenocyte activation could be detected, albeit the magnitude of this activation was small. In the presence of Con-A, immobilized mAb CL21 augmented the splenocyte mitogen response. Although the enhancement of the Con-A response suggests that CL21 may be involved in T-cell activation, the augmentation we observed could also be due to recruitment of an additional population of cells that did not respond to the mitogen. Thus, the conditions of the present experiments did not address the specific cell type that is responsive to mAb CL21.

Neither splenocyte activation nor enhancement of the Con-A response was observed when mAb CL21 was present in suspension. The requirement that mAb CL21 must be bound to a substrate in order to effect activation is consistent with the notion that the natural ligand may be either on the surface of other cells or a component of the extracellular matrix.

The molecular mechanisms involved in lineage specification within the hematopoietic system are not known. Recent experiments using human bone marrow indicate that the 180-kD isoform of the CD45 antigen (CD45RO) is expressed on more primitive, clonogenic hematopoietic progenitors, including the primitive erythroid progenitors BFU-e and precursors able to initiate long-term bone marrow cultures (Lansdorp et al., 1990). In contrast, most CFU-gm express the 220-kD isoform, CD45R. In *Xenopus*, mRNA for the $\beta$ chain of larval hemoglobin ($\beta$T1) is detectable between stages 28 and 32 (Banville and Williams, 1985), the same period when CL21 expression is first detected. Because of its early developmental expression, the 180-kD CL21 antigen may be one of the first molecules transcribed following committment of unrestricted lateral plate mesoderm to hematopoiesis and is most likely present on pluripotential hematopoietic stem cells. This observation invites speculation that the CL21 determinant may be an accessory component of signal transduction complex involved in specifying cell lineage within the hematopoietic system. By analogy with CD45 and T-cell signal transduction, CL21 may modulate signals originating from an array of receptors present on the surface of primitive hematopoietic precursors.

### MATERIALS AND METHODS

### Animals

Gravid female *Xenopus* were initially obtained from commercial suppliers (Xenopus I, Ann Arbor, MI; Carolina Biological Supply, Burlington, NC) and have been repeatedly bred in the laboratory. All animals used in these studies were derived from outbred embryos fertilized in the laboratory and reared under optimal conditions. Postmetamorphic juveniles were at least 6 months of age.

### Preparation of Monoclonal Antibodies

*Immunogens.* Peripheral blood and thymuses were removed from juvenile frogs, washed, and stored at 4°C over night in amphibian phosphate-buffered saline (APBS) containing 0.5% BSA. Plasma membranes were prepared according to the dextranpolyethylene glycol aqueous two-phase system of Brunette and Till (1971). Briefly, 50 g of 20% dextran (w/w); 25.75 g of 30% PEG (w/w); 24.75 ml distilled water; 83.25 ml of 0.22 M phosphate buffer, pH 6.5; and 20 ml of $10^{-2}$ M $ZnCl_2$ were mixed in a separatory funnel and allowed to separate at 4°C. Cell suspensions were washed once in APBS, suspended in $10^{-3}$ M $ZnCl_2$, and incubated 30 min at 4°C. Cells were gently ruptured on ice using a 7 ml Dounce homogenizer with pestel A (800–1000 strokes). The resulting homogenate was pelleted (10,000 X G) and suspended in 1.5 ml of the top phase of the separating system. 1.5 ml of the bottom phase was added, the phases were mixed and centrifuged at 10,000×G for 10 min in a fixed-angle rotor using an IEC model B200 high-speed centrifuge. The plasma membranes, confirmed by electron microscopy of erythrocyte preparations (data not shown), were concentrated at the interface between the two phases and the nuclei were found in the pellet. The interface was collected, diluted fourfold with distilled water, and the membranes were pelleted by an additional centrifugation at 10,000×G for 10 min. The final membrane pellet was suspended in mammalian saline.

***Immunization.*** In order to tolerize mice to *Xenopus* erythrocyte cell-surface determinants, 3-month-old Balb/c mice were injected subcutaneously with purified membranes from $10^8$ erythrocytes emulsified in complete Freund's adjuvant (CFA). Three days following the immunization, mice were injected with 150 mg/kg cyclophosphamide, delivered in three equal doses at 2-day intervals (Matthew and Patterson, 1983). One week after the last injection, mice were subcutaneously immunized with membranes from 5× $10^7$ thymocytes emulsified in CFA. Mice were boosted at biweekly intervals for 2 months with thymocyte membranes emulsified in incomplete Freund's adjuvant. Three days prior to fusion, mice were injected intraperioteneally with thymocyte membranes suspended in PBS.

***Fusion.*** Draining lymph nodes were fused with NS1 plasmocytomas using standard protocols. Fusion, initial culturing of hybridomas, and cloning by limiting dilution were done in the University of Nebraska Medical Center Monoclonal Antibody Facility.

***Screening.*** Hybridomas were screened by flow cytometry beginning at 10 days postfusion. Mixtures containing $10^5$ thymocytes and $10^5$ erythrocytes were incubated in hybridoma supernatant diluted to amphibian osmolarity, washed two times, incubated in FITC labeled goat anti-mouse Ig, and analyzed for green fluorescence using an Ortho 50 H flow cytometer. The eliptical shape of frog erythrocytes enabled us to established gates such that one region contained predominantely thymocytes with a 15% RBC contamination and a second region contained mostly erythrocytes. The initial screening was based on the presence of a positive signal in the thymocyte window coupled with the absence of signal in the erythrocyte window. Selected wells were cloned three times by limiting dilution. The selected hybridoma, CL21, secretes an $IgG_{2ak}$ antibody.

### Immunoprecipitation

Cell-surface radiolabeling of *Xenopus* hematopoietic cells with $^{125}I$ followed by immunoprecipitation and protein analysis were carried out according to published methods (Flajnik et al., 1984; Kauffman et al., 1985). Briefly, surface molecules were labeled using lactoperoxidase catalized iodination. Labeled cells were lysed in 0.5 ml buffer consisting of 20 mM Tris-HCl, 0.15 M NaCl, 1 mM $MgCl_2$, and 2% NP-40. For each immunoprecipitation, $10^6$ cell equivalents were precleared three times with 15 $\mu l$ of a 50% suspension of Protein A-sepharose, incubated overnight with the ascites from either CL21 or XL-1 and precipitated with Protein A-sepharose. Sepharose beads were washed three times in protein-A wash buffer (50 mM Tris-HCl, pH 8.0; 5 mM EDTA; 650 mM NaCl; 0.5% Triton-X-100, v/v; 1 mg/ml ovalbumin), three times in low-salt buffer (50 mM Tris-HCl, pH 8.0; 5 mM EDTA; 150 mM NaCl; and 0.5% Triton-X-100) and boiled 4 min in Laemmli sample buffer (Laemmli, 1970). Both reduced and nonreduced samples were run on 5–15% $NaDodSO_4$ polyacrylamide gels.

### Affinity Purification

A BioRad Affi-gel Protein A MAPS kit was used according to manufacturer's instructions. Eluted fractions were dialyzed against APBS and analyzed for protein content using the spectrophotometric method of Groves et al. (1968).

### Tissue Culture

***Embryonic mesoderm.*** Stage 20 embryos were placed in sterile 100% DeBoer's solution (DBS, 0.11 M NaCl, 1.3 mM KCl, 4 mM $CaCl_2$, pH 7.2, with $NaHCO_3$) containing 1% pen-strep (10,000 U/ml–10 mg/ml). Jelly coats and vitelline membranes were removed using watchmaker's forceps. Embryos were passed through ten changes of 10% DBS containing 1% pen-strep. Lateral plate mesoderm was removed from 20-hr-old embryos and transferred to 96 well microtiter plates containing dilute L-15 media (200 milliosmole) supplemented with $1.25\times10^{-2}$ M

$NaHCO_3$, Fe-Transferrin (28 μg/ml), $5\times10^{-5}$ M 2-ME, and 1% pen-strep, and gassed with 5% $CO_2$ in air. Explants were cultured for periods ranging from 1 to 10 days as previously described (Turpen and Smith, 1985).

*Metabolic activation.* The MTT (3-[4,5-Dimethylthiazol-2-yl]-2,5-diphenyltetrazolium bromide) assay described by Mossman (1983) was modified for use in a solid-phase assay with amphibian cells. Complete details of the development of this protocol will be published elsewhere. Briefly, goat antimouse IgG (10 μg/ml) (TAGO, Inc.) was immobilized onto 96 well flat bottom microtiter plates (Falcon) according to Wysocki and Sato (1978). After blocking the plates with 5% BSA, varying concentrations of test antibodies (CL21, 76-2-11, XT2.1) were added and incubated 2–4 hr at 4°C. Splenocytes, $1–2\times10^5$ per well, were plated in RPMI 1640 with $NaHCO_3$ (diluted to 220 milliosmoles) and containing 10 mM Hepes, 1% pen-strep Glutamine (Irvine Scientific), 1 mM Sodium Pyruvate, $5\times10^{-5}$ M 2ME, 28 μg/ml Fe-Transferrin (Sigma, Human Transferrin), 120 μg/ml delipidated, deionized BSA, and 2 nM phorbal-myristate acetate. Concentrations of Con-A ranged from 0 to 10 μg/ml. Final culture volume was 100 μl per well. Cultures were maintained at 26°C in 5% $CO_2$ in air. After 48 hr, 10 μl of MTT (5 mg/ml in APBS) was added to each well. At 72 hr, the assay was developed by adding 200 μl of acidified (0.04N HCl) isopropanol and incubating at room temperature for 30 to 60 min or until all crystals were dissolved. The assay was read on a Skatron Eliza Reader with a reference wavelength of 620 nm and a test wavelength of 570 nm. Data are expressed as change in optical density calculated by subtracting unstimulated from stimulated optical-density values.

### Immunofluorescence

*Cytology.* Cell suspensions from embryos and mesodermal cultures as well as larval and adult hematopoietic organs were collected with a cytocentrifuge (Shandon Southern), air dried 3 min, fixed in 70% EtOH in DBS at 4°C for 5 min, postfixed in 95% EtOH for 2 min, and stored in a dessicator. Prior to antibody staining, slides were rehydrated with APBS containing 0.5% BSA and incubated overnight at 4°C with either 50 μl or a 1:50 dilution of ascites or 100 μl of tissue culture supernatant. Slides were washed three times in APBS, incubated for 1 hr with FITC-labeled goat antimouse IgG $Fab_2'$ (20 μg/ml) (TAGO, Inc.), washed three times, and mounted in 10% glycerol in APBS. Stained preparations were examined using epifluorescence and a Zeiss IM 30 inverted microscope equipped with a 50-W mercury light source.

*Histology.* Adult tissue was snap frozen by embedding in Tissue Tek O.T.C. compound (Baxter Scientific) followed by immersion in an isopentane bath containing dry ice. Specimens were stored at –70°C prior to sectioning. Ten micron sections were cut with a cryostat, fixed in acetone, and stained as before.

*Flow cytometry.* For cell-surface immunofluorescence, $10^5$ live cells were incubated 45 min with 100 μl culture supernatant, 1/100 dilution of ascites or 20 μg/ml affinity-purified antibody, washed twice at 4°C in APBS containing 0.5% BSA plus 0.5% sodium azide (ABA), incubated for 30 min in FITC-labeled goat antimouse IgG $Fab_2'$ (20 μg/ml) washed twice, and analyzed using a Ortho 50 H flow cytometer equipped with an argon laser, set at 488 nm with an output of 150 mW. Data were collected and analyzed using Cicero (Cytomation Inc., Boulder, CO).

For cell sorting, $10^6$ splenocytes were incubated in 100 μl ABA containing 20 μg/ml affinity-purified antibody and stained as before. CL21 positive cells were sorted from CL21 negative cells on the basis of fluorescence intensity using a Becton Dickinson FACStar$^{plus}$. The morphology of sorted cells was examined using cytospin preparations followed by Giemsa or PAS staining. The PAS reaction was performed according to Hayhoe and Quaglino (1988). Briefly, cytospins were fixed with formalin vapor followed by consecutive incubations in periodic acid and Schiff's leucobasic fuchsin.

### Antibodies

The following antibodies with indicated specificities were used: mAb XT2.1, anti *Xenopus* T cell (Nagata, 1985); mAb XL 1, anti *Xenopus* leukocyte (Ohinata et al., 1989); mAb AM20, anti *Xenopus* MHC class II (Flajnik et al., 1990); mAb 6.16, anti *Xenopus* IgM (Bleicher and Cohen, 1981); mAb 76-2-11, antiswine cytotoxic T cell (Isotype control, ATCC #HB143); unconjugated goat antimouse IgG and FITC-conjugated goat antimouse IgG F(ab)$_2$' (TAGO).

### ACKNOWLEDGMENTS

We thank Drs Ch. Katagiri and H. Ohinata for providing us with the XL-1 antibody, Dr. D. A. Crouse for the 76-2-11 hybridoma, and Dr. Martin Flajnik for the gift of AM20 and XT2.1 hybridomas. This research was supported by grants HD-18566 from the USPHS and DCB-9017516 from the National Science Foundation.

(*Received January 21, 1991*)

(*Accepted May 16, 1991*)

### REFERENCES

Banville D., and Williams J.G. (1985). Developmental changes in the pattern of larval β-globin gene expression in *Xenopus laevis*. Identification of two early larval β-globin mRNA sequences. J. Mol. Biol. **184:** 611–620.

Bleicher P.A., and Cohen N. (1981). Monoclonal anti-IgM can separate T-cell from B-cell proliferative responses in the frog, *Xenopus laevis*. J. Immunol. **127:** 1549–1555.

Brunette D.M., and Till J.E. (1971). A rapid method for the isolation of L-cell surface membranes using an aqueous two-phase polymer system. J. Membrane Biol. **5:** 215–224.

Du Pasquier L., Schwager J., and Flajnik M.F. (1989). The immune system of *Xenopus*. Ann. Rev. Immunol. **7:** 251–275.

Flajnik M.F., Kauffman J.F., Riegert P., and Du Pasquier L. (1984). Identification of class I major histocompatibility complex encoded molecules in the amphibian *Xenopus*. Immunogenetics **20:** 433–442.

Flajnik M.F., Soldano F., Cohen N., and Du Pasquier L. (1990). Evolution of the MHC: Antigenicity and unusual tissue distribution of *Xenopus* (frog) class II molecules. Mol. Immunol. **27:** 451–462.

Groves W.E., Davis F.C., Jr., and Sells B.H. (1968). Spectrophotometric determination of microgram quantities of protein without nucleic acid interference. Analyt. Biochem. **22:** 195–210.

Hadji-Azimi I., Coosemans V., and Canicatti C. (1987). Atlas of adult *Xenopus laevis* hematology. Dev. Comp. Immunol. **11:** 807–874.

Hayhoe F.G.J., and Quaglino D. (1988). Haematological cytochemistry, 2nd ed. New York: Churchill Livingstone, p. 82.

Kauffman J.F., Flajnik M.F., and Du Pasquier L. (1985). *Xenopus* MHC class II molecules: I. Identification and structural characterization. J. Immunol. **134:** 3248–3257.

Laemmli U.K. (1970). Cleavage of structural proteins during the assembly of the head of the bacteriophage T4. Nature **227:** 680–687.

Lansdorp P.M., Sutherland H.J., and Eaves C.J. (1990). Selective expression of CD45 isoforms on functional subpopulations of CD34$^+$ hemopoietic cells from human bone marrow. J. Exp. Med. **172:** 363–366.

Ledbetter J.A., Tonks N.K., Fischer E.H., and Clarke E.A. (1988). CD45 regulates signal transduction and lymphocyte activation by specific association with receptor molecules on T or B cells. Proc. Natl. Acad. Sci. USA **85:** 8628–8632.

Lefrancois L., and Goodman T. (1987). Developmental sequence of T200 antigen modifications in murine T-cells. J. Immunol. **139:** 3718–3724.

Matthew W.D., and Patterson P.H. (1983). The production of a monoclonal antibody that blocks the action of a neurite outgrowth-promoting factor. Cold Spring Symp. Quant. Biol. **48:** 625–631.

Mossman T. (1983). Rapid colorimetric assay for cellular growth and survival: Application to proliferation and cytotoxicity assays. J. Immunol. Methods **65:** 55–63.

Nagata S. (1985). A cell-surface marker of thymus-dependent lymphocytes in *Xenopus leavis* is identifiable by mouse monoclonal antibody. Eur. J. Immunol. **15:** 837–841.

Nieuwkoop P.D., and Faber J. (1967). Normal table of *Xenopus laevis* Daudin Amsterdam: North-Holland.

Ohinata H., Tochinai S., and Katagiri Ch. (1989). Ontogeny and tissue distribution of leukocyte-common antigen bearing cells during early development of *Xenopus laevis*. Development **107:** 445–452.

Pingle J.T., and Thomas M.L. (1989). Evidence that the leukocyte-common antigen is required for antigen-induced T lymphocyte proliferation. Cell **58:** 1055–1065.

Samelson L.E., Fletcher M.C., Ledbetter J.A., and June C.H. (1990). Activation of tyrosine phosphorylation in human T-cells via the CD2 pathway. Regulation by the CD45 tyrosine phosphatase. J. Immunol. **145:** 2448–2454.

Smith S.H., Brown M.H., Rowe D., Callard R.E., and Beverley P.C.L. (1986). Functional subsets of human helper–inducer cells defined by a new monoclonal antibody, UCHL1. Immunology **58:** 63–70.

Thomas M.L. (1989). The leukocyte common antigen family. Ann. Rev. Immunol. **7:** 339–369.

Tonks N.K., Charbonneau H., Diltz C.D., Fischer E.H., and Walsh K.A. (1988). The demonstration that the leukocyte common antigen CD45 is a protein tyrosine phosphatase. Biochemistry **27:** 8695–8701.

Turpen J.B., and Smith P.B. (1985). Dorsal lateral plate mesoderm influences proliferation and differentiation of hemopoietic stem cells derived from ventral plate mesoderm during early development of *Xenopus laevis* embryos. J. Leu. Biol. **38:** 415–427.

Wysocki L.J., and Sato V.L. (1978). Panning for lymphocytes: A method for cell selection. Proc. Natl. Acad. Sci. USA **75:** 2844–2848.

